# Pallidal neurostimulation versus botulinum toxin injections in the treatment of cervical dystonia: protocol of a randomized, sham-controlled trial (StimTox-CD)

**DOI:** 10.1186/s42466-019-0007-3

**Published:** 2019-02-28

**Authors:** Thorsten M. Odorfer, Uwe Malzahn, Cordula Matthies, Peter U. Heuschmann, Jens Volkmann

**Affiliations:** 1 Department of Neurology, University Hospital Würzburg, Josef-Schneider-Str. 11, 97080 Würzburg, Germany; 2 Clinical Trial Center, University Hospital Würzburg, 97080 Würzburg, Germany; 3 Department of Neurosurgery, University Hospital Würzburg, 97080 Würzburg, Germany; 4 Institute of Clinical Epidemiology and Biometry, University of Würzburg, Comprehensive Heart Failure Center, University of Würzburg, 97080 Würzburg, Germany; 5 Department of Neurology, University Hospital Würzburg, 97080 Würzburg, Germany

**Keywords:** Cervical dystonia (CD), Pallidal neurostimulation, Deep brain stimulation (DBS), Botulinum neurotoxin type a (BoNT a), Motor symptom control, TWSTRS (Toronto Western spasmodic torticollis rating scale), dystonia-associated quality of life, Safety

## Abstract

**Background:**

Selective peripheral denervation via botulinum neurotoxin injections into dystonic muscles is the first-line treatment for cervical dystonia. Pallidal neurostimulation is a potent alternative, but currently restricted to patients failing on neurotoxin therapy. As botulinum neurotoxin is partially effective but often unsatisfactory in a relevant proportion of patients, earlier neurostimulation might be advantageous in providing stable symptom control and preventing disability. This trial intends to demonstrate, that pallidal neurostimulation is superior to neurotoxin injections in best clinical practice for controlling the symptoms of cervical dystonia and that it is safe in patients with a partial therapy response to peripheral denervation. We hypothesize a better outcome in everyday functioning and health-related quality of life of neurostimulated patients.

**Methods:**

We aim to recruit 66 cervical dystonia patients into a double-blind comparison of pallidal neurostimulation versus botulinum neurotoxin type A. Eligible patients need ≥25% motor symptom reduction 4 weeks after a neurotoxin test injection, but are willing to undergo DBS surgery due to unsatisfactory symptom control. All participants will be implanted with a DBS system, and randomized into 2 groups: First group will receive effective neurostimulation and saline injections into dystonic muscles. Second group is treated with regular neurotoxin injections and undergoes a sham-stimulation. Primary outcome is the change in TWSTRS total score between baseline and 6 months of therapy. Secondary outcome parameters are corresponding changes in TWSTRS motor score, Tsui score, CDQ-24 and SF-36. Safety will be assessed by frequency and severity of reported adverse events. Statistical analysis includes intention-to-treat and per protocol populations, analysis based on imputation of missing values and analysis adjusting for differences in baseline TWSTRS. After 6 months of blinded treatment all patients will receive open-label neurostimulation and neurotoxin treatment as needed, and are followed up 48 weeks after randomization.

**Perspective:**

We will assess if pallidal neurostimulation is a safe and effective alternative to selective peripheral denervation by botulinum toxin injections in cervical dystonia, which may be offered earlier in the course of disease based on patient preference. A positive study outcome would influence future treatment guidelines of cervical dystonia.

**Trial registration:**

EudraCT registration number:  2016–001378-13

## Background

Cervical dystonia (CD) is a central nervous system disease associated with involuntary muscle contractions, leading to repetitive or constant neck movements and partly bizarre head postures [1]. CD is estimated to be the most frequent of focal dystonias [9]. Activities of daily living, social participation and quality of life can be markedly impaired in CD due to pain, reduced active head and neck motion ranges as well as stigmatization (Camfield, Ben-Shlomo, Warner, & Epidemiological Study of Dystonia in Europe Collaborative, [4, 15]).

Chemical denervation via injection of botulinum neurotoxin (BoNT) into dystonic neck muscles is applied as first line treatment of CD; its efficacy and safety has been demonstrated in several randomized controlled trials [5, 18]. BoNT injections are shown to reduce mean symptom severity substantially defined as reduction between 9.7 to 11 points on Toronto Western Torticollis Rating Scale (TWSTRS) [2, 6]. In addition, its application is superior to pharmacological treatment with anticholinergic drugs [3]. As the neurotoxin effect is only temporary, repeated injections are necessary within a period of 8 to 16 weeks. Furthermore, the toxin effect builds up within a period of 1–4 weeks after injection; therefore, CD symptom severity tends to fluctuate during BoNT therapy. Beside these pharmacological response fluctuations, patients may experience a variable benefit from repeated injections, because the treatment is skill-dependent and limited by the reproducibility of applying equivalent toxin dosages into partly deep lying neck muscles. Beyond the principle efficacy established in pivotal trials, few studies have addressed the long-term efficacy and stability of BoNT treatment responses in daily practice. In an observational study Misra et al. reported that more than 60% of CD patients are treated satisfactorily with BoNT; however, considering the criterion of “duration of effect ≥ 12 weeks” reduced this proportion up to 29% [14]. A further limitation of BoNT therapy arises when complex dystonic patterns including tremor or jerky movements appear; in these cases, many muscles are involved in an intricate interplay, which apparently impedes a target-oriented injection. Additionally, dose-dependent adverse events (AE) may limit the treatment, such as neck weakness, dysphagia, dry mouth or dysphonia [7]. During chronic treatment, secondary therapy failure due to BoNT neutralizing antibodies develops in up to 5% of patients [10]. Despite these limitations and the discomfort associated with repeated BoNT injections, many patients adhere to this therapy, because they are not aware of potential alternatives.

Deep brain stimulation (DBS) is a surgical therapy for movement disorders, which retunes abnormal neuronal activity within motor circuits by continuous electrical stimulation of deep brain nuclei. DBS requires the surgical implantation of stimulating electrodes connected to a pacemaker device. There is class I evidence for the safety and efficacy of DBS applied to several brain targets (subthalamic nucleus, internal globus pallidus (GPi) and thalamus) for patients with Parkinson’s disease (PD) [8], tremor disorders [17] and dystonia [12]. Treatment effects of DBS are strong and can lead to almost complete symptom suppression even in severely disabled patients. Nevertheless, this therapy has been restricted to advanced disease stages after failure of pharmacological treatment alternatives out of principle safety considerations. DBS surgery carries a risk of permanent morbidity and mortality resulting from intracranial haemorrhage or infections, while the treated conditions are disabling but not life limiting by themselves. Otherwise, DBS may come too late in advanced disease stages for restoring function and societal participation in chronically disabled patients despite being effective on a symptom level. Improved surgical techniques and perioperative management have now reduced the chronic morbidity and mortality risk of DBS below 0.5% [11], which has allowed considering earlier DBS with the goal to maintain function and prevent psychosocial decline in PD. For example, the EARLYSTIM trial [16], a controlled randomized comparison of subthalamic DBS with best medical management of PD with early motor fluctuations has established superiority of DBS for improving off-period symptoms, response fluctuations and quality of life. Interestingly, there was no difference in the total number of AE and serious AE between both treatment groups over the 2 year study period, suggesting that the risks associated with medical treatment or inappropriate symptom control of PD have been largely underestimated in the past and may be mitigated by DBS.

GPi-DBS is an established first line therapy for patients with generalized dystonia, for whom no pharmacological treatment alternatives exist [12, 19]. Previous trials have also established efficacy and safety in patients with CD refractory to neurotoxin therapy. In a severely affected study population, a mean reduction of 5.2 points (26%) on TWSTRS motor and 18.3 points (40%) on TWSTRS total score was achieved by GPi-DBS [19]. Additionally, also pain, activities of daily living and quality of life are shown to improve during DBS treatment (Kupsch et al., 2006; [19, 20]).

Based on this evidence, we believe, that the acceptable safety profile and the therapeutic potency of DBS justify an earlier application in CD patients still responding to peripheral selective denervation, but suffering from incomplete symptom control or response fluctuations. In this trial we aim to prove that GPi-DBS leads to a superior symptom control in comparison to best clinical use of BoNT in the group of CD patients with a partial response to BoNT therapy. Consequently, we also hypothesize that dystonia-associated quality of life improves more with DBS treatment. Additionally, safety of DBS will be explored in this patient group.

## Methods/design

### Study protocol

**Fig. 1:**
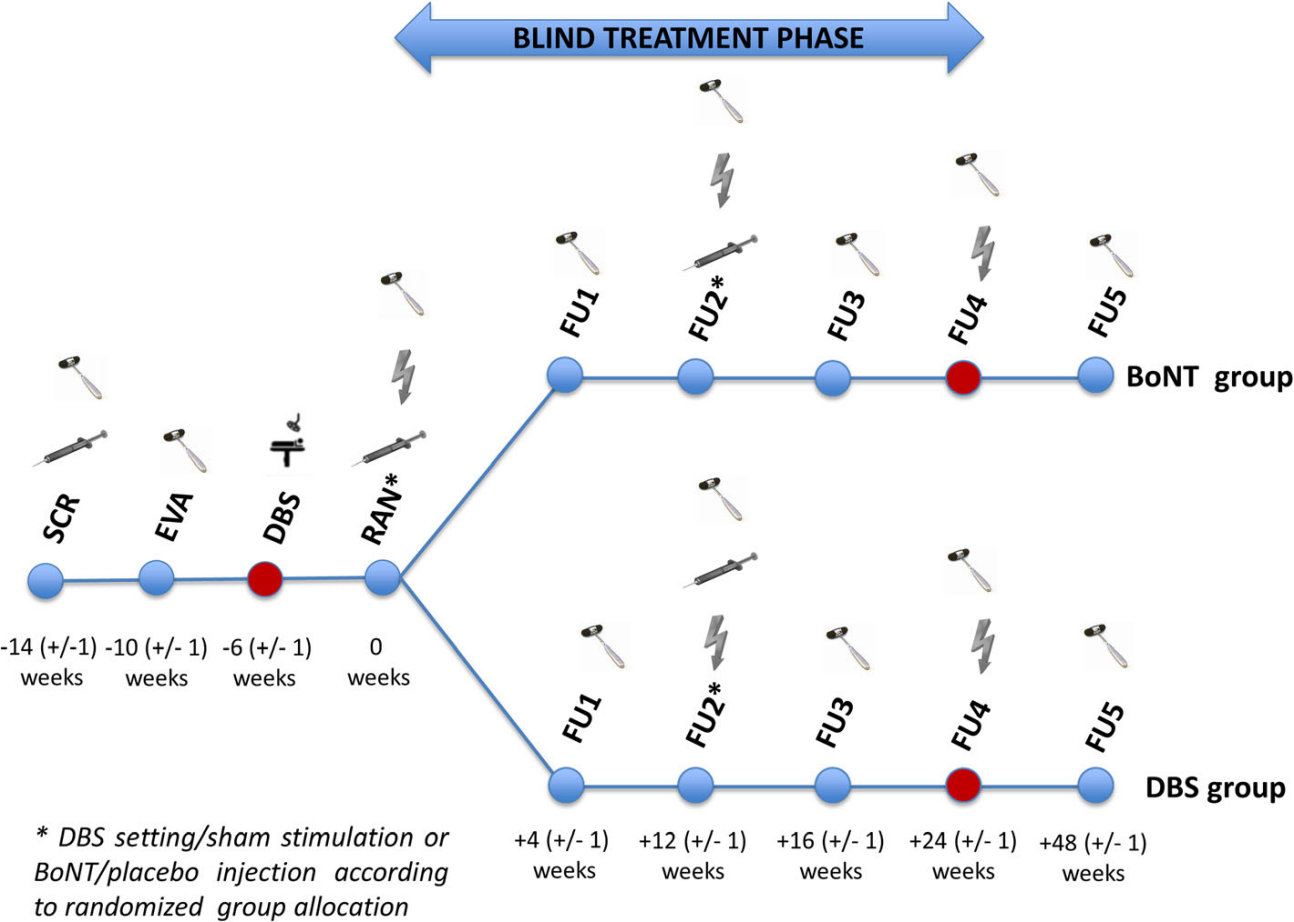
Visit schedule of StimTox-CD: SCR = screening visit, EVA = evaluation visit, DBS = DBS surgery, RAN = randomization visit, FU = follow-up visit, icon “syringe” ≙ BoNT or placebo injection, icon “reflex hammer” ≙ clinical assessment, icon “operating room” ≙ DBS surgery, icon “lightning” ≙ DBS setting/sham stimulation

Figure 1 shows the study schedule of StimTox-CD. During “screening visit” (SCR) a test BoNT injection is applied to prove partial therapy response of neurotoxin treatment. 4 (±1) weeks later in “evaluation visit” (EVA) treatment effect is quantified by clinical assessment comprising inter alia TWSTRS motor score. Finally, a minimal motor-symptom reduction of 25% is required to fulfil all inclusion criteria. Table 1 shows a comprehensive list of the criteria applied to choose eligible patients for the study.

**Table 1:** Eligibility criteria

Inclusion criteria	Exclusion criteria
• Suffering from isolated, idiopathic or hereditary cervical dystonia for more than 2 years • Moderate to severe symptom severity of dystonia (TWSTRS total score ≥ 20 points and TWSTRS motor score ≥ 15 points) • Age between 18 and 75 years • Adequate therapy response of BoNT A treatment during test injection procedure (≥ 25% reduction of points in TWSTRS motor score 4 weeks after baseline) • Previous BoNT injection ≥12 weeks before screening visit • Written informed consent to study participation, including patient’s agreement to undergo DBS procedure	▪ Mild dystonic symptoms (TWSTRS total score < 10 points and TWSTRS motor score < 5 points) ▪ Suffering from severe depression with ongoing suicidality (BDI > 25 points) ▪ Mild cognitive impairment (MCI) and dementia (MDRS ≤125 points) ▪ Ongoing acute psychosis ▪ Drug or alcohol abuse ▪ Pregnancy or lactation, women of childbearing age without secure contraception ▪ Illiteracy ▪ Surgical contraindication to DBS ▪ Concurrent participation in other clinical trials ▪ Any medical or psychological condition associated with the risk of insufficient compliance and/or early termination of the study

All participants undergo stereotactic implantation of deep brain electrodes in GPi under general anaesthesia. Comparable to previous dystonia DBS studies, the applied neurostimulation system is ACTIVA PC (MEDTRONIC, Minneapolis, USA), including matching electrodes 3387 and 3389 [13, 19]. DBS procedure depends on local standards in each study centre. There are no detailed specifications on perioperative management and stereotactic procedure. Only a preoperative MRI-scan (including T1-, T2, gadolinium-enhanced and inversion recovery sequences) in general anaesthesia and a postoperative image control of electrode positioning are mandatory.

6 (±1) weeks after DBS implantation patients are divided in two study arms during “randomization visit” (RAN). Table 2 is showing the full data set, which is collected at RAN as baseline for the upcoming blinded study phase. Neurostimulation will be now activated for the first time and acute stimulation effects and adverse effects of all electrodes are tested in a structured fashion (“monopolar review”). In DBS group a local DBS expert implements an effective stimulation setting concerning stimulation amplitude and choice of electrodes after “best clinical practice” and a predetermined algorithm. Frequency must be adjusted to 130 Hz, pulse duration to 90 μs. In BoNT group stimulation amplitude is set to 0 V. This group is injected with BoNT by an experienced physician. The decision on preferred drugs (Onabotulinumtoxin A: BOTOX, Abobotulinumtoxin A: DYSPORT or Incobotulinumtoxin A: XEOMIN), applied dosage and injection technique (number of injections, injection site or guiding-techniques) is up to the discretion of the treating physician and supposed to correspond to best clinical practice. During the same visit, patients in DBS group get also injected, but only with saline. Neither patient nor treating physician is informed about the allocation to the study group, in the sense of a double-blind trial design. Obviously DBS setting is not appropriate to be implemented in a blind manner, what causes the need of two separate teams of physicians at each study site; one blinded (or BoNT) and one unblinded (or DBS) team.

**Table 2:** Synopsis of interventions and data sets (displayed for single SimTox-CD study visits)

	Screening visit/SCR	Evaluation visit/EVA	DBS surgery/DBS	Randomization visit/RAN	Follow-up visit 1/FU1	Follow-up visit 2/FU2	Follow-up visit 3/FU3	Follow-up visit 4/FU4	Follow-up visit 5/FU5
timeline (weeks)	−14 (+/−1)	− 10 (+/− 1)	−6 (+/− 1)	0	+4 (+/− 1)	+ 12 (+/− 1)	+ 16 (+/− 1)	+ 24 (+/− 1)	+ 48 (+/− 1)
Interventions									
BoNT test injection	X								
DBS surgery			X						
Testing of stimulation (side) effects				X					
DBS setting/sham stimulation^a^				X		X			
Blind BoNT/placebo injection^a^				X		X			
Unblind neurostimulation								X	X
Randomization				X					
Data set									
Informed consent	X								
Inclusion/exclusion criteria	X	X							
Medical history	X								
Concurrent medication	X				X	X	X	X	X
TWSTRS	X	X		X	X	X	X	X	X
Tsui Rating Scale	X	X		X	X	X	X	X	X
Bain Tremor Rating	X	X		X	X	X	X	X	X
GCI	X	X		X	X	X	X	X	X
CDQ-24	X			X				X	X
SF-36	X			X				X	X
EQ-5D	X			X				X	X
MDRS	X			X				X	(X)
BDI	X			X				X	(X)
BPRS	X			X				X	(X)
Video recording	(X)	(X)		X	(X)	(X)	(X)	X	X
Questionnaire on DBS procedure			X						
Postoperative control of electrode position				X					
AE					X	X	X	X	X
DBS settings				X	X	X	X	X	X

^a^according to randomized group allocation

Abbreviations (in alphabetical order): *AE* adverse events, *BoNT* Botulinum neurotoxin, *BDI* Beck Depression Inventory, *BPRS* Brief Psychiatric Rating Scale, *CDQ*-24 Craniocervical Dystonia Questionnaire, *DBS* deep brain stimulation, *EQ-5D* Euroquol (life quality questionnaire), *GCI* Global Clinical Impression Scale, *MDRS* Mattis Dementia Rating Scale, *TWSTRS* Toronto Western Spasmodic Torticollis Rating Scale, *SF*-36 Short Form 36 (life quality questionnaire)

4 (±1) weeks after randomization first follow-up visit (FU1) is scheduled. Clinical outcome parameters are evaluated by blinded physicians. The same procedure is repeated during FU2 and FU3 (12 (±1) and 16 (±1) weeks after RAN), with a second blindly applied BoNT or placebo injection. In FU2 also adjustments of DBS settings are allowed to improve neurostimulation effect in DBS group. The blinded study phase ends in FU4, 24 (±1) weeks after randomization. Neurostimulation is now activated also in BoNT group and the open trial phase starts. Up from FU4, patients are treated commonly without restrictions concerning DBS programming or BoNT injection, treatment must basically conform again to best clinical practice. During FU5, after another interval of 24 (±1) weeks, study ends and a last detailed clinical assessment is intended.

## Outcome measures

The primary outcome parameter is the difference in TWSTRS total score 6 month after implantation. We hypothesize that under optimized treatment in both groups there should be no discrepancy in symptom severity at FU3 (4 weeks after injection of BoNT at the time of medication’s peak effect).

We defined changes in TWSTRS motor score, TSUI score, CDQ-24 and SF-36 to serve as secondary outcome parameters. Additionally, we measure frequency and severity of spontaneously reported AE in both treatment arms.

Additionally, we will describe the difference in TWSTRS total score between patients of DBS group at FU4 and BoNT group at FU5. At both time points, patients are treated with effective neurostimulation for 6 months, respectively. We suppose the same therapy response of effective DBS in both arms, regardless if the stimulation is applied in a blinded or unblinded fashion.

As other hypothesis we will investigate if the maximum treatment effect of DBS is achieved 6 month after activation. Therefore, we will quantify variations in TSWTRS score in the DBS group 6 and 12 months after randomization.

Motor symptom severity is basically evaluated by using standardized and well-established scales like TWSTRS motor and Tsui score. Tsui includes also a tremor item, which is missing in TWSTRS. Dystonic head tremor is also assessed by the Bain Tremor Rating Scale. Additionally, the severity of dystonia is ranked by patients and physicians using a numeric rating scale (1–10) as element of the Global Clinical Impression (GCI) scale.

Questionnaires referring to pain and disability are also part of TWSTRS total score. Another dystonia-specific tool applied to assess quality of life of patients suffering from cervical dystonia is the Craniocervical Dystonia Questionnaire (CDQ-24). Two further standardized forms supposed to measure general health-related quality of life were chosen to be completed by study participants: SF-36 (Short Form 36) and EQ-5D (Euroquol). To exclude relevant cognitive deficits Mattis Dementia Rating Scale (MDRS) will be applied. Depressive symptoms are assessed by Beck Depression Inventory (BDI). For other psychiatric comorbidities we screen by using the Brief Psychiatric Rating Scale (BPRS).

All scales and questionnaires are used in their German version.

### Randomization

Patients are allocated in a 1:1 ratio into the two study arms by stratified block randomization. Randomization procedure is operated centrally via GCP-compliant EDC system. The categorized baseline TWSTRS total score serves as single stratification factor to aim approximately equal distribution of symptom severity in both groups.

### Statistics

Sample size calculation is based on primary outcome parameter TWSTRS total score change from baseline to FU4. The primary statistical analysis consists in a mean value comparison of the primary outcome between both treatment groups by the independent two-sample T-Test. For sample size calculation we refer to data from previously published studies. A GPi-DBS trial showed a mean therapy effect in TWSTRS total score in patients undergoing neurostimulation of minus 23.1 (±15.2 SD) points [19]. A mean maximum effect of neurotoxin treatment is described with minus 11.0 (±11.7 SD) points on TWSTRS total score at an average of 4 weeks after injection [6]. The resulting standardized mean difference of TWSTRS scores between both groups is 0.90. Using these assumptions a sample size of 2 × 28 = 56 patients ensures a power of 90% to detect this effect size as significant (at significance level 0.05) deviation from the null hypothesis (no difference between mean TWSTRS change scores in DBS and BoNT group). Taking into account a drop-out rate of 15% (conservative estimation) 2 × 33 = 66 must be randomized. Furthermore we assume that 20% of patients will refuse informed consent to undergo DBS after screening and another 20% will fail inclusion criteria of sufficient botulinum treatment response after test injection. Consequently, a total number of 2 × 52 = 104 patients will have to be screened to reach the calculated sample size.

Group comparisons for target variables with sample distributions compatible with the assumption of normality will be conducted by using the two-sided independent sample Student’s T-Test or the paired sample T-Test for within group comparisons (changes between visits). For variables with sample distributions not compatible with the assumption of normality non-parametric tests like Mann-Whitney-Test, Wilcoxon Signed Rank Test or Friedmann Test will be applied. Effects will be considered significant if *p* < 0.05.

The primary statistical analysis of the primary outcome parameter as well as the secondary outcome parameter change in TWSTRS motor score, TSUI score and SF-36 score will be based on the intention-to-treat (ITT) population. To proof robustness of results two further analyses of sensitivity will be performed: the first one refers to all randomized patients with imputation of missing values after 6 months by appropriate methods of missing-data imputation depending on which missing-data mechanism could be reasonable. The second method includes only patients of per protocol (PP) population.

To address the question how much TWSTRS total score values differ between DBS and BoNT group on average, when patients would be start at baseline with the same score values, we will use ANCOVA (target variable: TWSTRS total change score from baseline to 6 months after operation, main factor: treatment method, covariate: baseline TWSTRS). Thereby we will adjust to imbalances in the TWSTRS distributions at baseline and the variance within the groups should be reduced.

Also for secondary outcome parameters an ANCOVA will be applied using treatment group as main factor, adjusted for each baseline variable as covariate. Furthermore longitudinal data of scores will be analysed by using GEE (generalized estimating equation) and linear models (“mixed models”). This will be helpful to describe interindividual variability of therapy response.

Safety analysis contains a structured list of AE of both study groups. Possibility of relation to study’s interventions will be judged by physicians of the unblinded team. Incidence of AE will be assessed by using Fisher’s Exact Test (analysis by patients: comparisons of proportions of patients with at least one corresponding event) and negative binomial regression (analysis by events: comparisons of counts of occurring events).

## Perspective

StimTox-CD is the first multicentre, randomized, double-blind and sham-controlled trial of DBS vs. best conservative therapy, specifically selective peripheral denervation with BoNT in CD patients, which have a partial treatment effect of ongoing neurotoxin injections. The study hypothesizes that pallidal neurostimulation is more effective in controlling CD symptoms than BoNT treatment and that is safe in this indication, too.

### Visit schedule

To provide a fair comparison between the two treatments with time varying efficacy, we took special care in scheduling the study visits. Baseline visit takes place 6 weeks after operation. This interval was chosen to reduce the impact of the surgical stun effect of electrode implantation. Additionally, motor symptom control efficacy is evaluated in FU1 and FU3 (each 4 weeks after BoNT injection), when the neurotoxin treatment effect is supposed to be maximal [6].

### Quality management

In order to maintain comparable and highest treatment standards at all study sites, we only recruited DBS centres operating more than 25 patients within a year. All 11 participating sites are university hospitals in Germany with a longstanding experience in DBS and a BoNT outpatient clinic run by an expert physician (board certified neurologist and special experience in neurotoxin treatment).

Furthermore, we provide a controlled rating training for TWSTRS score to increase inter-rater-reliability. To reduce possible bias trough changing or different examiners, a standardized video recording is scheduled at RAN, FU4 and FU5 to enable a central evaluation of symptom severity.

### Blinding

Two independent teams at each site are established to guarantee that the study could be performed in a blinded fashion. The BoNT reconstitution process must be conducted by an unblinded team member. Provided in neutral syringes, solutions of BoNT or saline are not distinguishable from each other, as both having no special colour or smell.

DBS settings are mandatory to be implemented with stimulation amplitude of 0.5 V below the threshold of possible adverse effects, which was tested before during monopolar review process. This should ensure that patients are not able to perceive switch-on of DBS system.

### Summary

To the best of our knowledge, StimTox-CD is the first randomized, double-blind and controlled comparison of DBS and BoNT in CD. As described, the study protocol is designed to answer the question which therapy method is more effective in controlling motor symptom severity and provides better outcomes concerning disease-related disability and quality of life. The fact that patients will be included in this trial, which have a partial but not satisfactory treatment effect of neurotoxin injections, addresses the unmet medical need of alternative treatment strategies for this less disabled CD population. A positive study outcome would have impact on treatment guidelines for CD. On the condition that DBS proves to be safe in this indication, physicians might be able to offer DBS earlier in the course of CD and empower patient treatment choices.

## Abbreviations

AE: adverse events
BDI: Beck Depression Inventory
BoNT: botulinum neurotoxin
BPRS: Brief Psychiatric Rating Scale
CD: cervical dystonia
CDQ-24: Craniocervical Dystonia Questionnaire
DBS: deep brain stimulation
EQ-5D: Euroquol (life quality questionnaire)
EVA: Evaluation visit
FU: Follow up visit
GDRS: Global Dystonia Rating Scale
GEE: Generalized estimating equation
GPi: Globus pallidus internus
ITT: Intention-to-treat population
MCI: Mild cognitive impairment
MDRS: Mattis Dementia Rating scale
PD: Parkinson’s disease
PP: per protocol population
RAN: randomization visit
SCR: screening visit
SD: standard deviation
SF-36: Short Form 36 (life quality questionnaire)
TWSTRS: Toronto Western Spasmodic Torticollis Rating Scale
